# Coronary artery calcification is not associated with survival in advanced gastric and gastroesophageal junction cancer: a secondary analysis of the EXPAND trial

**DOI:** 10.1007/s00432-026-06654-y

**Published:** 2026-11-03

**Authors:** Hans-Jonas Meyer, Tihomir Dermendzhiev, Paul-Philipp Jacobs, Daniele Romeo, Gertraud Stocker, Timm Denecke, Florian Lordick, Ulrich T. Hacker

**Affiliations:** 1 Department of Diagnostic and Interventional Radiology, University Hospital Leipzig, Leipzig, 04103 Germany; 2 Department of Biomedicine, Neuroscience and Advanced Diagnostics (BiND), University of Palermo, Palermo, Italy; 3 Department of Medicine II, University Cancer Center Leipzig (UCCL), University of Leipzig Medical Center, Cancer Center Central Germany (CCCG), Leipzig, 04103 Germany

**Keywords:** Advanced gastric cancer, Computed tomography, Coronary calcification score

## Abstract

**Purpose:**

Computed tomography (CT) is the clinical standard for staging of gastrointestinal cancer. Previous studies have highlighted the prognostic significance of coronary artery calcification (CAC) detected by CT in various types of cancer. However, the prognostic role of CAC scoring in gastrointestinal cancers is still unclear. The aim of this study was to investigate the impact of CT-defined CAC on overall survival (OS) in patients with advanced gastric or gastroesophageal junction cancer.

**Methods:**

CAC was semiquantitatively measured using the visual Weston method on baseline CT scans in 785 patients with unresectable locally advanced or metastatic gastric or gastroesophageal junction cancer from the phase III EXPAND trial. This present study is a secondary analysis of the base line CT images. In the original analysis patients were randomly assigned to first-line treatment with cisplatin/capecitabin ± cetuximab. The primary endpoint of the current subanalysis is the OS. For statistical analysis group differences were calculated using the Mann-Whitney-U test and ANOVA test. Kaplan-Meier curves and multivariable Cox regression analysis were used to test the effect of CAC on mortality.

**Results:**

The median CAC score was 1 (IQR 0; 3). A total of 337 patients (42.9%) had no visible CAC (CAC score 0). Male patients had statistically significant higher CAC score compared to female patients (median 1 versus 0, *p* < 0.0001). In univariable Cox regression CAC showed no significant association with survival when analysed as a continuous value (HR: 0.99, 95% CI: 0.96–1.02, *p* = 0.74), and when dichotomized for absence and presence of calcification (HR:1.08, 95% CI: 0.92–1.26, *p* = 0.34). Similar results were identified in further subgroup analyses.

**Conclusion:**

CT-defined CAC scoring has no relevant prognostic impact in advanced gastric and gastroesophageal junction cancer undergoing first-line chemotherapy. The present study cannot confirm the promising results for CAC scoring in lung cancer patients.

## Introduction

Irresectable, locally advanced and metastatic gastric and gastric-esophageal junction (GEJ) cancer prognosis remains poor and treatment options continue to be limited despite recent advances. Despite the development of new chemotherapy regimens, the 5-year survival of these patients in the first-line chemotherapy setting is still less than 10%, and therefore a high and unmet need exists for efficacious treatment (Choo et al. 2026, Hultman et al. 2017).

Regarding prognostic factors, tumor-centric factors such as TNM stage and histopathological features have been used as the primary determinants of prognosis (Arigami et al. 2025, Dixon et al. 2016). A growing interest of oncological research focus on patient-related factors and comorbidities, which can also have a relevant impact on the overall treatment outcomes and overall survival of oncological patients (Götze et al. 2025, Mais et al. 2021, Meyer et al. 2022, Choi et al. 2024). In particular, cardiovascular status and general body composition parameters are sometimes underestimated as factors in determining the prognosis for various types of cancer. Furthermore, patients with gastrointestinal cancer tend to be older, have a history of smoking and suffer from obesity, all of which are established risk factors for cardiovascular diseases and atherosclerosis (Kapoor et al. 2020).

One of the important cardiovascular risk factors defined by computed tomography (CT) is coronary artery calcification (CAC) (McConachie et al. 2022, Agatston et al. 1990, Kirsch et al. 2012). Besides the current gold standard, the Agatston score, which is still based on cardiac-synchronized CT scans, the semiquantitative Weston score offers a simple and practical alternative for quantifying CAC in oncological staging CT scans (Kirsch et al. 2012).

Promising recent studies suggest that CAC scoring provides new prognostic information for oncological care, which was especially shown for lung cancer patients (Meyer et al. 2025, Osawa et al. 2020). In lung cancer, CAC scoring was associated with overall survival with a reported hazard ratio of 1.42 95% CI (1.19; 1.69) in a meta analysis based upon 2292 patients (Meyer et al. 2025). However, systematic results are still lacking for most other tumor entities. In esophageal cancer, it was shown that the extent of CAC progresses within 2 years after the initiation of radiotherapy, particularly in patients with detectable CAC before radiotherapy initiation (Matsuo et al. 2023). Notably, this study investigated squamous cell cancer patients with a history of smoking as a risk factor, comparably to the risk profile of lung cancer patients (Matsuo et al. 2023). A recent study on gastric and colorectal cancer patients demonstrated that patients without CAC had a significantly better oncological outcome than high burden patients with an Agatston score over 400 (Kim et al. 2025). Therefore, additional data are needed on cardiovascular risk profiling using CT images in order to further validate their utility as prognostic markers for oncological outcomes and thereby improve patient care.

The EXPAND trial was a randomized phase 3 trial investigating patients with locally advanced unresectable or metastatic gastric cancer or adenocarcinomas of the GEJ. Patients receiving first-line chemotherapy with or without cetuximab. The trial enrolled patients in several countries worldwide (Lordick et al. 2013). This study population provides a relevant data set to test the prognostic relevance of CAC scoring in advanced tumor stages and could be adjusted for ethnical differences.

Since the prognostic significance of CAC scoring in these tumor entities has hardly been studied to date (Kim et al. 2025, Xue et al. 2026), further analysis is needed to understand diagnostic and prognostic value of a semiquantitative CAC scoring using the CT images of the patients.

Therefore, the present study elucidates the associations between CAC scoring defined by baseline CT images with overall survival in advanced gastric and gastroesophageal junction cancer patients undergoing first-line chemotherapy using the EXPAND RCT dataset.

## Methods

### Patient acquisition

The EXPAND trial included patients with unresectable, locally advanced, or metastatic gastric or GEJ cancer. The aim of the study was to demonstrate a survival benefit for the addition of the anti-epidermal growth factor receptor antibody cetuximab to standard chemotherapy (capecitabine/cisplatin). Specifically, in this open‐label, randomized, controlled, phase 3 study, adults with histologically confirmed adenocarcinoma of the stomach or GEJ with locally advanced unresectable (M0) or metastatic (M1) disease were enrolled at 164 sites in 25 countries worldwide (Lordick et al. 2013).

**Fig. 1:**
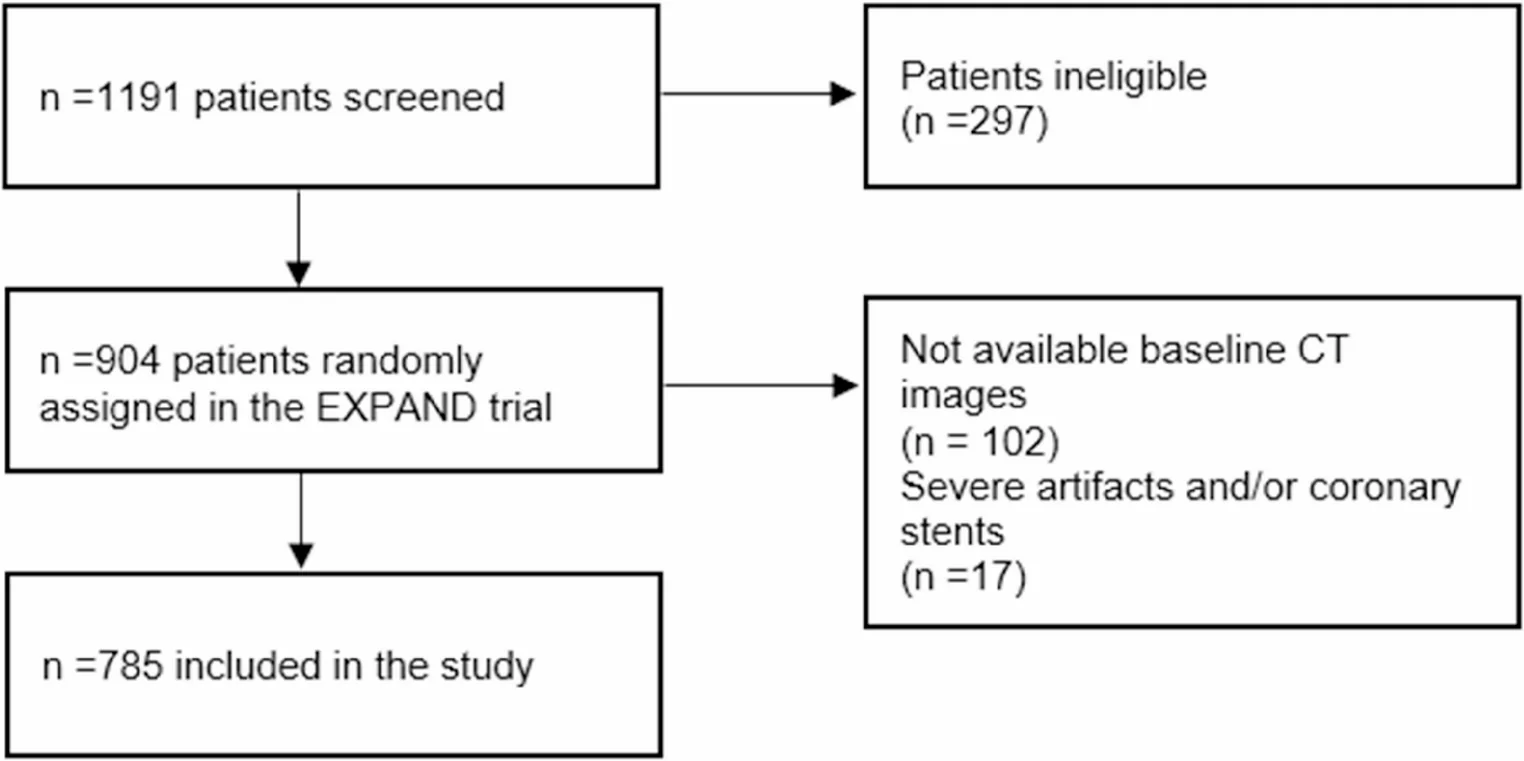
Flow chart of the patient cohort acquisition. A total of 785 patients were included in the present study

Since there were no statistically significant differences in PFS and OS between treatment arms, all patients were included in this retrospective analysis. A total of 904 patients were randomized for treatment. Of these, 785 baseline CT scans (86.8%) were evaluable for the present analysis of CT parameters. The reason for lacking images is that in rare cases magnetic-resonance imaging was performed for staging purposes and some CT scans were not available for central reading. In 17 cases severe imaging artifacts and or implanted coronary stents led to the exclusion of the analysis. A previous secondary analysis explored the prognostic relevance of CT-defined body composition using this data set (Hacker et al. 2022). Figure 1 provides the flow chart of the acquisition of the patient cohort.

The study was performed according to the principles of the Declaration of Helsinki, and translational research was approved by local/national ethics committee. This present secondary analysis for this study was approved by the Ethics Committee of the Medical Faculty, Leipzig University, Leipzig, Germany (code 044/26-ek).

### CAC scoring

All patients underwent contrast enhanced CT imaging using multidetector scanners with a slice thickness of ≤ 5 mm for clinical staging. Digital Imaging and Communications in Medicine (DICOM) data were collected centrally. In all instances, the baseline CT images were used for this analysis. All CT images were re-analyzed retrospectively by a trained radiology resident with overall 3 years of experience using the institutional picture archiving and communication system. Two window settings bone (width 1800 level 400) and soft tissue window (width 350 level 50) were used to discriminate between contrast media and calcifications.

**Fig. 2:**
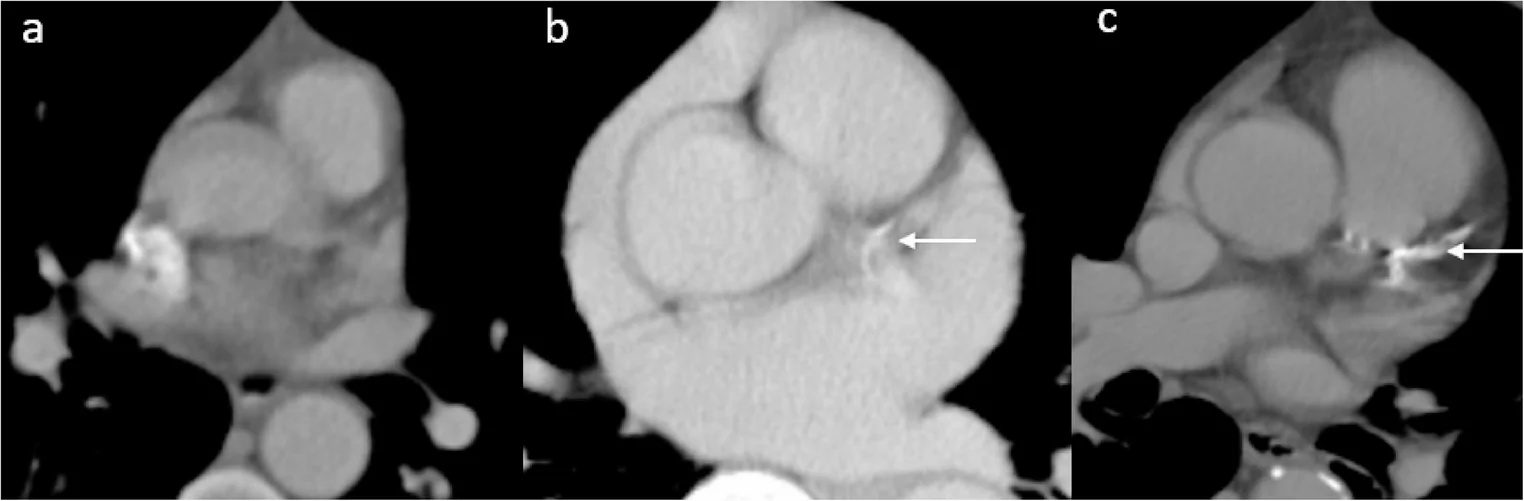
Representative case examples from the patient cohort. **A** A patient without visible calcification resulting in a Weston score of 0. **B** A case with small calcifications in the left anterior descending coronary artery resulting in a Weston score of 2. **C** A patient with extensive coronary calcifications in the left anterior descending coronary artery. The patient had a Weston score of 6

The CAC was measured with the Weston score, an established semiquantitative score ranging from 0 to 12 (Kirsch et al. 2012). The coronary arteries are divided into left main, left anterior descending, left circumflex and right coronary and every vessel can be scored on a 4-point scale. This score showed a high inter-reader agreement and a high correlation with the current diagnostic gold standard of the Agatston score in the initial description (Kirsch et al. 2012). In unclear cases with small hyperdense spots, the patients were scored as 0. Representative cases of the cohort are shown in Fig. 2.

### Statistical analysis

All statistical analyses were performed using GraphPad Prism 10 (GraphPad Software, La Jolla, CA, USA). and R (version 4.4.3) in ambient RStudio (version 2026.1.0.392, Posit team (2026). *RStudio: Integrated Development Environment for R*. Posit Software, PBC, Boston, MA. http://www.posit.co/). Differences among groups were assessed using Mann-Whitney-U-test and ANOVA test, when suitable. Continuous variables are presented as medians with interquartile ranges (IQR) and categorical variables as counts and percentages. Spearman’s r was used to perform correlations between the quantitative variables. Univariate and multivariate Cox proportional hazard regression were used to estimate hazard ratios (HR) and their corresponding 95% confidence intervals (CI) in univariable and multivariable analyses. Variance inflation factor (VIF) analysis was performed to assess multicollinearity among predictors. Survival time was calculated from the date of randomization to the date of death or last follow-up. Predictors reporting a p-value < 0.1 were included in the multivariable analysis. Kaplan-Meier curves were used to show the overall survival and log-rank test was used to compare the curves. For Cox regression analysis, the ordinal variable T stage was dichotomized into low (T0-2) and high (T3-4), N and M stage were dichotomized in 0 vs. positive. In all instances, statistical tests were two-sided and a value *p* < 0.05 was considered statistically significant.

## Results

### Patient characteristics

A total of 785 patients (200 female patients, 25.5%) with a median age of 59 years (range 18–84 years) were included in the present study. The demographics overview is given in Table 1. 4% of patients had locally advanced unresectable disease; all other patients had metastatic disease. 82% of patients suffered from gastric cancer, while 16.2% were diagnosed with GEJ cancer. Almost 40% of patients were from Asia, and 60.5% from non‐Asian regions of the world.

**Table 1:** Baseline characteristics of the study population

Characteristic	*N* (%) or Median and IQR
Study population	785
Age, years	59.0 (51.0–66.0)
Sex	
Female	200 (25.5%)
Male	585 (74.5%)
Ethnicity	
Asian	310 (39.5%)
White	415 (52.9%)
Hispanic	54 (6.9%)
Black	3 (0.4%)
Not specified	3 (0.4%)
Disease type	
Gastric adenocarcinoma	642 (81.8%)
AEG type I	43 (5.5%)
AEG type II	49 (6.2%)
AEG type III	39 (5.0%)
Not Determined	11 (1.5%)
T stage	
0	59 (7.5%)
1	7 (0.9%)
2	63 (8.0%)
3	230 (29.3%)
4	175 (22.3%)
Tx	251 (32.0%)
N stage	
0	74 (9.4%)
1	171 (21.8%)
2	139 (17.7%)
3	136 (17.3%)
Nx	265 (33.8%)
M stage	
0	21 (2.7%)
1	763 (97.2%)
Mx	1 (0.1%)
Weston Score	1.0 (0.0–3.0)
Score 0	337 (42.9%)
Positive score	448 (57.1%)
Score < 7	720 (91.7%)
Score 7 or higher	65 (8.3%)

*Abbreviations*: *AEG* Adenocarcinoma of the esophagogastric junction, *IQR* Interquartile range

**Fig. 3:**
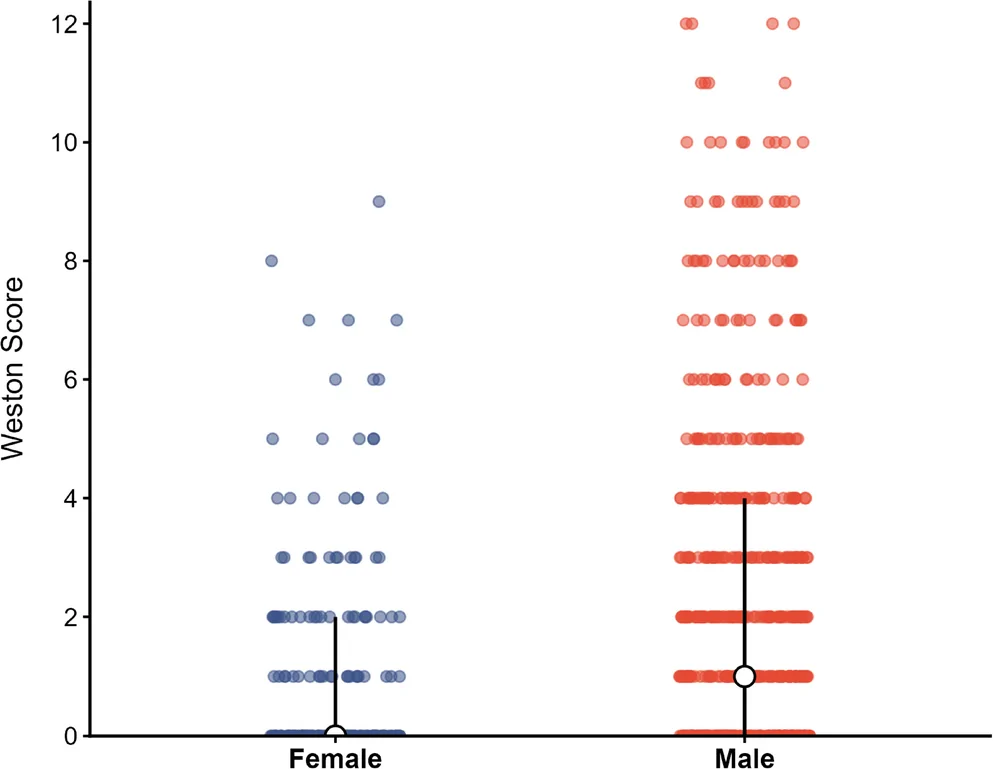
Scatter plot showing the differences between male and female patients in regard of CAC score. The male patients had significantly higher CAC score. (1 vs. 0; *p* < 0.0001)

**Fig. 4:**
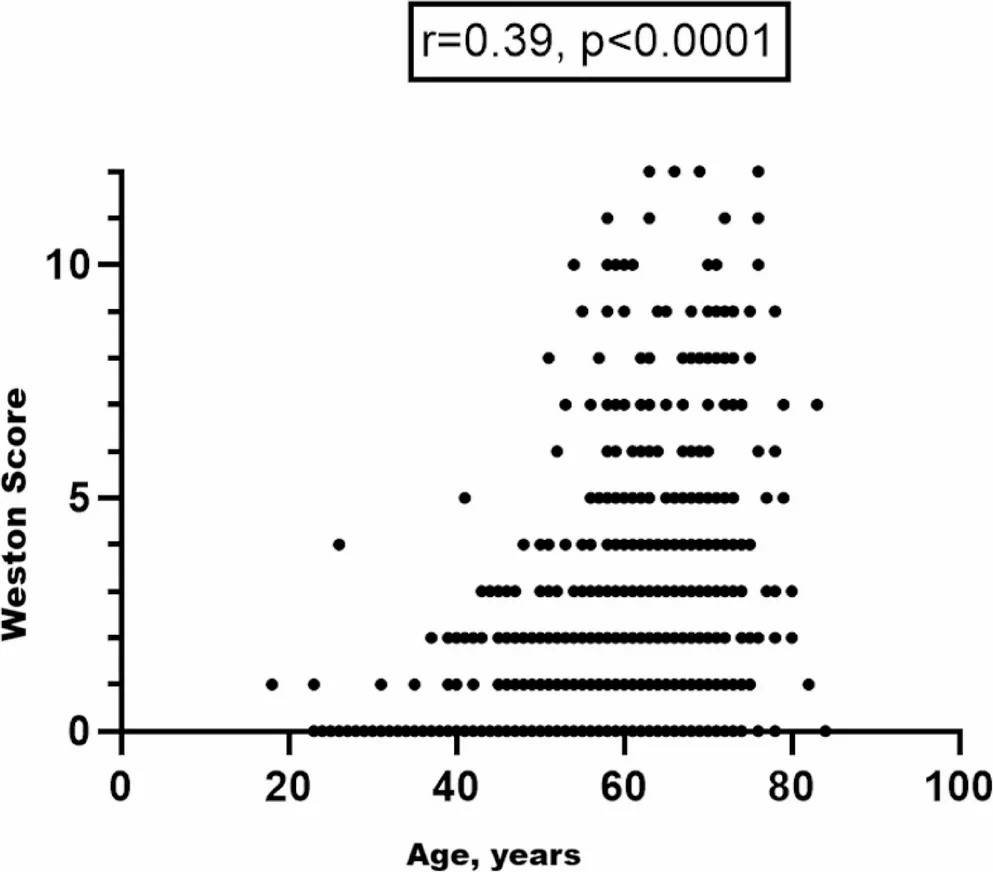
Spearman’s correlation demonstrating the association between age and CAC score (*r* = 0.39, *p* < 0.0001)

**Fig. 5:**
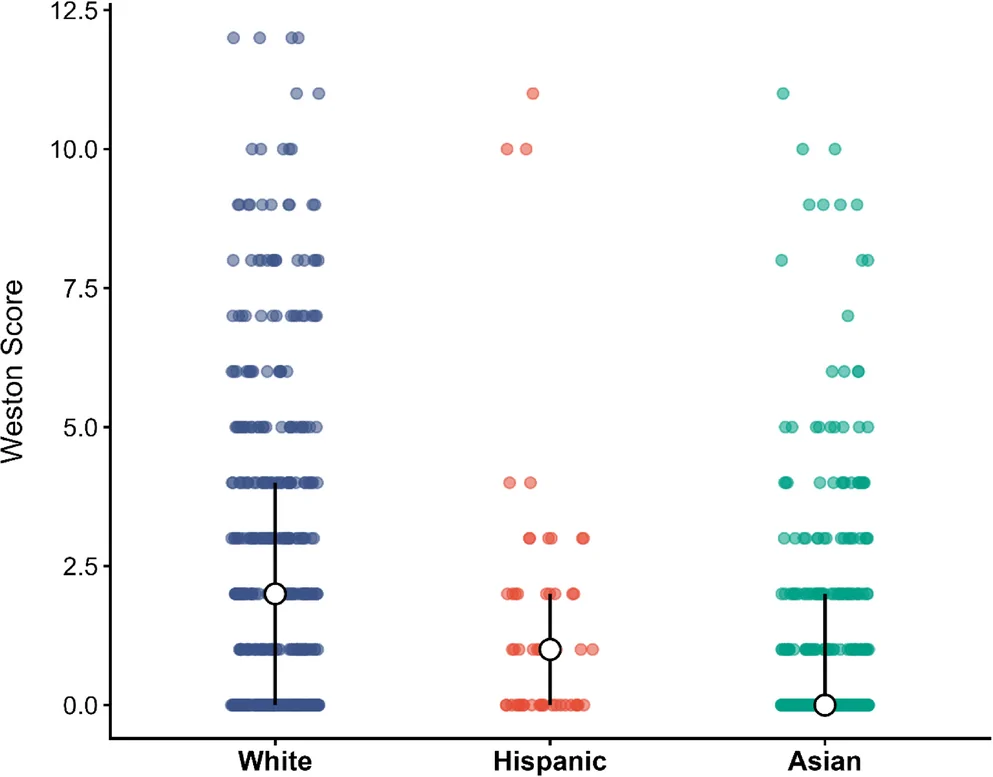
Differences of CAC score according to ethnicity. The white patients had the highest CAC score (ANOVA test *p* < 0.0001)

The median CAC score was 1 (IQR 0; 3), ranging from 0 to 12. A total of 337 patients (42.9%) had no visible CAC (CAC score 0). Male patients had statistically significant higher CAC score compared to the female patients (median 1 versus 0, *p* < 0.0001). The corresponding scatter plot is shown in Fig. 3. CAC showed a moderate positive correlation with age (*r* = 0.39, *p* < 0.0001) (Fig. 4). Regarding ethnicity, the white patients’ subgroup showed statistically significant the highest CAC score (median of 2), compared to Asian (median 0) and Hispanics (median 1), ANOVA test *p* < 0.0001 (Fig. 5). There was no correlation with T-, N- or M- stage (*r* = 0.01, *p* = 0.85; *r*=-0.05, *p* = 0.24; *r*=-0.05, *p* = 0.15, respectively).

### Univariable Cox regression analysis

In univariable Cox regression analysis of the whole study population, CAC showed no significant association with survival when analysed as a continuous value (HR: 0.99, 95% CI: 0.96–1.12, *p* = 0.74) and dichotomized between absence and presence of CAC (HR:1.07, 95% CI: 0.92–1.26, *p* = 0.34). The cut-off of 7 was used as a surrogate of severely calcified arteries but also did not harbour prognostic relevance (HR: 0.91, 95%CI 0.68;1.22, *p* = 0.54).

Among clinical variables, age was significantly associated with overall survival (HR: 0.99, 95% CI 0.98-1.0, *p* = 0.022) as well as sex (HR: 0.82, 95% CI: 0.69–0.98, *p* = 0.034), a higher T stage (T3 and T4) (HR:1.36, 95%CI: 1.01–1.82, *p* = 0.041) and Asian ethnicity (HR: 0.84, 95% CI: 0.72–0.99, *p* = 0.037). In contrast, N stage (*p* = 0.066) and M stage (*p* = 0.288) were not statistically significant associated with overall survival.

### Multivariable Cox regression analysis

In multivariable Cox regression including Weston Score (continuous), adjusted for age, sex, ethnicity (Asian vs. non-Asian), N stage, T stage, the Weston score showed no significant association with overall survival (HR: 0.99, 95%CI 0.95–1.04, *p* = 0.81). The results of the Cox regression analyses are shown in Table 2.

**Table 2:** Univariable and multivariable Cox regression analysis of overall survival

Predictor	Univariable Cox regression		Multivariable Cox regression	
	HR (95% CI)	*p* value	HR (95% CI)	*p* value
Ethnicity (Asian)	0.84 (0.72; 0.99)	0.037	0.74 (0.58; 0.93)	0.011
Sex (Male)	0.82 (0.69; 0.98)	0.034	0.71 (0.55; 0.93)	0.013
Age	0.99 (0.98; 1.00)	0.022	0.99 (0.98; 1.00)	0.260
T stage (T3/T4)	1.36 (1.01; 1.82)	0.041	1.40 (1.03; 1.91)	0.033
N stage (positive)	1.31 (0.98; 1.75)	0.066	0.87 (0.55; 1.36)	0.534
M stage	1.30 (0.80; 2.10)	0.288		
Weston Score (continuous)	0.99 (0.96; 1.02)	0.741	0.99 (0.95; 1.04)	0.814
Weston Score (positive)	1.08 (0.92; 1.26)	0.343		
Weston Score cutoff 7	0.91 (0.68; 1.22)	0.540		

*Abbreviations*: *HR* Hazard ratio, *CI* Confidence interval

### Kaplan-Meier survival analysis

**Fig. 6:**
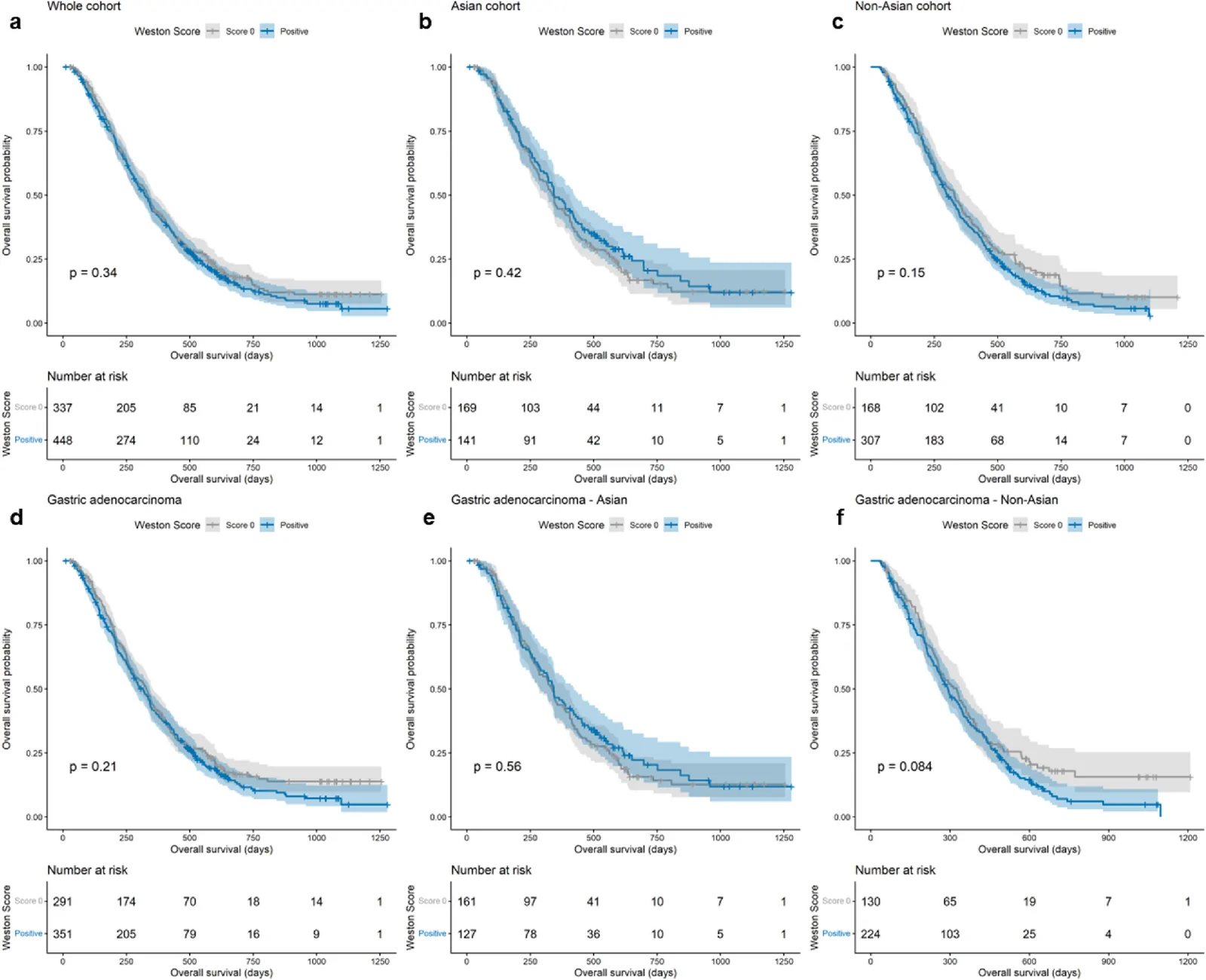
Kaplan Meier curves with subgroup analysis stratified for ethnicity between Asian and Non-Asian cohort

**Fig. 7:**
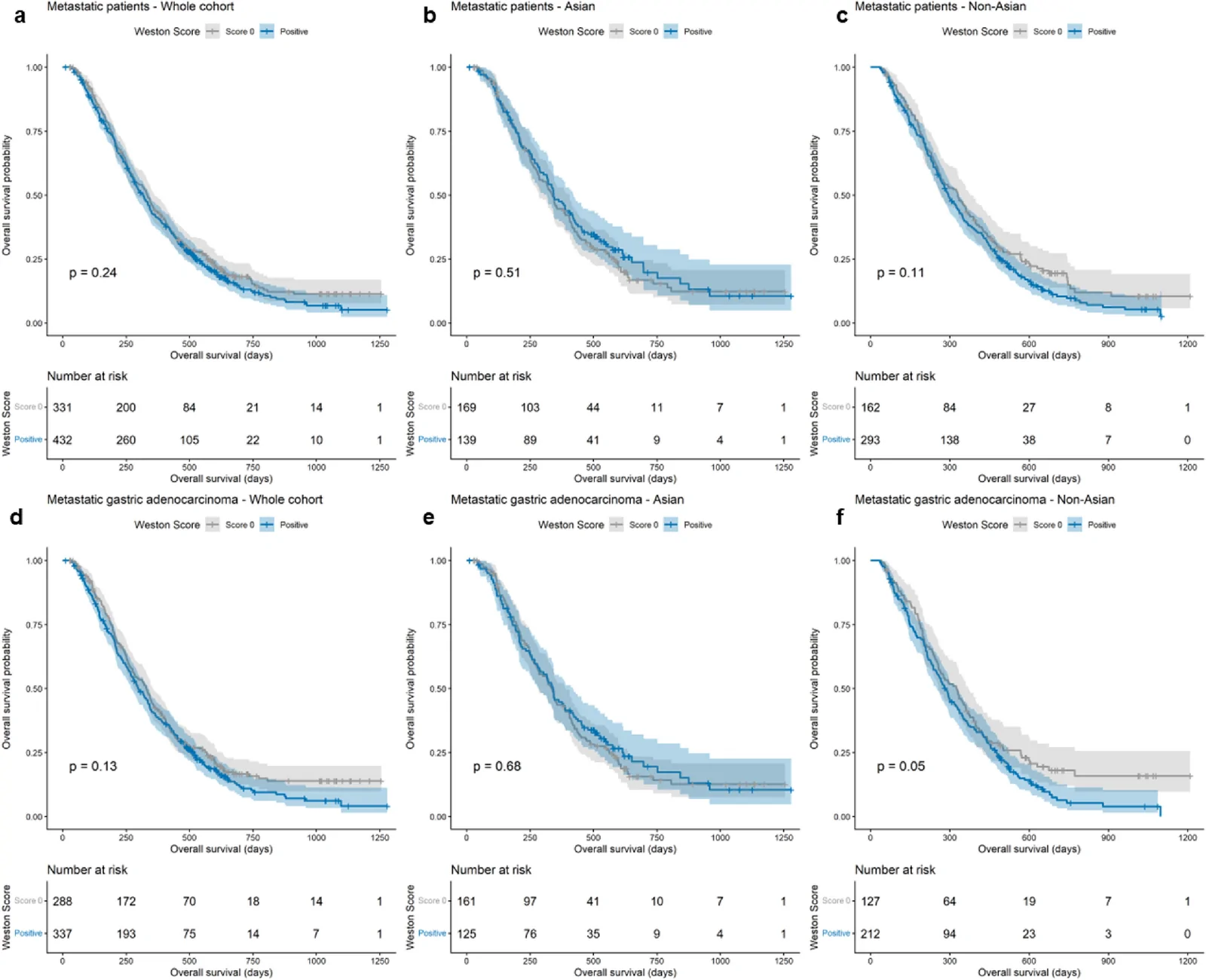
Kaplan Meier curves with metastatic disease-stratified subgroup analysis between whole population, Asian and Non-Asian cohort

Kaplan–Meier analysis demonstrated no stepwise association between CAC and reduced overall survival. When dichotomized for the presence or absence of CAC, patients with presence of CAC exhibited no shorter survival compared to those (log-rank *p* = 0.34). In the ethnicity-stratified subgroup analysis, higher CAC was not statistically significant associated with poor overall survival in any ethnic group. Among the patients with gastric adenocarcinoma, a trend toward shorter overall survival was shown in patients with higher CAC only in the Western cohort (log-rank *p* = 0.08). In the metastatic-stratified subgroup analysis, in western population, a trend toward shorter overall survival was shown in the subgroup with positive CAC score (log-rank *p* = 0.05). The corresponding Kaplan Meier curves are shown in Figs. 6 and 7.

## Discussion

This study demonstrates that CT-defined CAC scoring has no relevant impact on overall survival in patients with gastric and gastroesophageal junction cancer based upon a large international patient cohort from the randomized controlled EXPAND trial.

The current study is somewhat contradictory to recent exploratory studies highlighting the promising implication of comprehensive profiling of oncological patients defined by quantitative CT measures (Mais et al. 2021, Al-Sawaf et al. 2023). While CT-body composition parameters such as sarcopenia and visceral adiposity have been associated with adverse outcomes in gastrointestinal cancer with promising results (Götze et al. 2025, Meyer et al. 2022, Al-Sawaf et al. 2023, Wang et al. 2025), the prognostic relevance of CAC score is still unclear.

The role of CAC in thoracic cancer was recently summarized in a comprehensive review (Xue et al. 2026). The authors could provide frequencies of CAC with 26%, 66%, and 71% among patients with breast, esophageal (predominantly with squamous cell histology), and lung cancers, respectively (Xue et al. 2026). The current frequency of CAC of 42.9% is on the lower end of the published results, which may be explained by the high number of Asian patients with known lower CAC score compared to the Western population (McClelland et al. 2006).

One key finding of the review was that the evidence supporting the prognostic value of CAC for cardiovascular outcomes was strongest in breast cancer patients receiving adjuvant RT, whereas the results in lung and esophageal cancers were inconsistent (Xue et al. 2026). Beyond that, most studies focused on the prediction of major cardiovascular events rather than the survival aspects of the patients (Osawa et al. 2020, Xue et al. 2026).

For lung cancer, there was a clear association between the extension of CAC and survival outcomes as demonstrated in a recent meta-analysis (Meyer et al. 2025). This meta-analysis pooled results of overall 2.292 curatively treated lung cancer patients and showed a HR = 1.42 for the CAC score (Meyer et al. 2025). However, these results were mostly derived based on relatively small retrospective single center studies, thus limiting the evidence level.

For esophageal cancer (predominantly adenocarcinoma histology), a study could clearly demonstrate a significant association of CAC defined by CT images and major postoperative complications with an odds ratio of 4.04; 95% CI 1.34–12.16; *p* = 0.02 (Borggreve et al. 2018) in a cohort of patients treated with a curative intent. Despite these promising results, there was no previous survival analysis regarding CAC in esophageal cancer patients. In the same line, CT-defined aortic calcification was even associated with survival after curative resection in a small cohort of esophageal junction cancer patients (Hassan et al. 2026). Cardiovascular fitness appears to be of greater importance in patients who are undergoing curative treatment and may allow for better differentiation between patient groups (Xue et al. 2026). Another important aspect is related to the different risk profile that can be assumed depending on the histological type of esophageal cancer: in squamous cell cancer patients, risk profile the risk profile regarding smoking habits could well be comparable to that of lung cancer patients (Matsuo et al. 2023). In contrast, patients with adenocarcinomas may exhibit a different (i.e. lower) cardiovascular risk profile.

Only one study has investigated a similar patient sample using CAC scoring with the Agatston method (Kim et al. 2025). This retrospective study used a large gastric and colorectal cancer patient cohort of 3.153 patients and could demonstrate that patients without CAC had a significantly better outcome than high CAC burden patients with an Agatston score over 400. Notably, only 18.6% of the included patients were in stage IV and therefore were receiving palliative systemic treatment, when compared to the present study. Moreover, only 29.6% of patients had gastric cancer and the majority of cases suffered from colorectal cancer (Kim et al. 2025), again, limiting the comparability with the present study.

We could identify only a moderate association between CAC score and age, although the overall CAC burden is predominantly defined by the age (Duelli et al. 2025). In this large population study using cardiac CT, age and sex were the most influential predictors of the CAC burden, accounting for 69% and 18% relative predictor importance, respectively (Duelli et al. 2025).

In other oncology cohorts with multiple myeloma and different hematological diseases, CAC scoring was also not associated with overall survival, similar to the present study (Meyer et al. 2026, Meyer et al. 2025). Even a recent study using an AI algorithm investigating different tumor entities undergoing immune checkpoint inhibitors, no association between CAC burden and overall survival was identified (Noureldine et al. 2026).

Kirsch et al. showed that a Weston score > 7 is comparable to an Agatston score > 400 with sensitivity of 100%, and specificity of 98% (Kirsch et al. 2012), which we tested in further subgroup analyses. However, even for this patient group with highly calcified vessels, no significant survival differences were observed in our patient cohort. Presumably, the included patients in a randomized controlled trial have a better overall constitution compared to a real-world clinical cohort (Arigami et al. 2025) as investigated by Kim et al. (Kim et al. 2025). However, these differences need to be further analyzed in the future.

As an oncological study, the EXPAND trial did not include a systematic assessment of the cardiovascular risk profile of the patients (Lordick et al. 2013). This secondary analysis therefore cannot provide exploratory association analyses between the cardiovascular risk profile determined by CT and clinical characteristics.

Our results indicate that in advanced gastric cancer, cancer-associated factors such as tumor load and wasting carry greater prognostic relevance than markers of chronic cardiovascular comorbidity.

The present analysis has several limitations to address. First, it is a secondary analysis of a multicentric randomized controlled trial. The resulting bias due to the retrospective reading of the CT should be minimal as it was performed in a blinded fashion to the clinical results. Second, the resulting reader bias for this semiquantitative CAC scoring is small as demonstrated by the initial description (Kirsch et al. 2012). Third, due to the study design - which was typical for an oncology study - we were unable to correlate the CAC score with the clinical gold standard, the Agatston score derived from cardiac-gated CT images. However, the currently used Weston score has a high correlation with the Agatston score and was considered as a well-established surrogate score (Kirsch et al. 2012).

Fourth, one important aspect is that the initial study of the Weston score and most studies using this score relied on non-contrast enhanced CT images (Kirsch et al. 2012). In the present study using contrast enhanced staging CTs, very small calcifications could be mistaken with contrast media inside the vessel lumen. However, recent studies also used contrast enhanced CT images for opportunistic CAC scoring using the Weston method and AI algorithm (Meyer et al. 2026, Noureldine et al. 2026). To overcome this limitation, the measurement was performed in bone and soft tissue windowing to better discriminate between calcification and contrast media.

Fifth, while the EXPAND trial relied on chemotherapy plus or minus cetuximab and did not show statistical differences between treatment arms, in the meantime, treatment including immune checkpoint inhibition is standard of care for most patients, and other targeted approaches have become available to complement chemotherapy (Choo et al. 2026). It remains unclear, whether CAC scoring has prognostic impact in patients undergoing state of the art modern systemic treatment, which needs to be elucidated in the future.

## Conclusion

CT-defined CAC scoring has no association with overall survival in advanced gastric and gastroesophageal junction cancer undergoing first-line chemotherapy.

## Data Availability

No datasets were generated or analysed during the current study.
